# Peripheral T-cell lymphoma complicated by immunoglobulin A pemphigus: A case report and literature review

**DOI:** 10.3892/ol.2014.2088

**Published:** 2014-04-25

**Authors:** LINGJUAN CHEN, BOHAN YANG, JIQUAN FAN, KUNYU YANG, HONGLI LIU, GANG WU

**Affiliations:** Cancer Center, Union Hospital, Tongji Medical College, Huazhong University of Science and Technology, Wuhan, Hubei 430022, P.R. China

**Keywords:** T-cell lymphoma, autoimmune disease, pemphigus, thalidomide

## Abstract

Peripheral T-cell lymphomas (PTCLs) account for 12% of non-Hodgkin’s lymphomas (NHLs). Immunoglobulin (Ig) A pemphigus is an autoimmune blistering disease characterized by tissue-bound and circulating IgA antibodies that target epidermal cell surface components. Malignant lymphomas are often linked with autoimmune disease and the autoimmune blistering disease, paraneoplastic pemphigus, has been associated with NHL. However, cases of PTCLs that are complicated by IgA pemphigus are particularly rare. The current study presents the first known case of PTCL complicated by IgA pemphigus. A 43-year-old male was admitted to the Union Hospital (Wuhan, China) in March 2012 with multiple swollen lymph nodes. Pathology examinations revealed PTCL. Immunohistochemical staining was positive for cluster of differentiation (CD)2, CD3, CD5, CD7 and CD47, and negative for CD20. Ki-67 was ~40% positive. The patient was treated with four cycles of cyclophosphamide, Adriamycin, vincristine and prednisone, and two cycles of gemcitabine, cisplatin and dexamethasone; in addition, the patient received radiation of the retroperitoneal region (total dose, 36 Gy). The patient underwent thalidomide maintenance therapy for 20 days before flaccid blisters appeared on the trunk and limbs. Histopathology and immunofluorescence indicated IgA pemphigus, and intravenous methylprednisolone was administered, followed by treatment with prednisone. Subsequently, no evidence of recurrent lymphoma or pemphigus has been observed.

## Introduction

Peripheral T-cell lymphomas (PTCLs) are a diverse group of mature neoplasms that account for 12% of non-Hodgkin’s lymphomas (NHLs) (1). They are a rare and heterogeneous group of disorders that are predominantly associated with a poor prognosis (2). The standard PTCL treatment is administration of cyclophosphamide, doxorubicin, vincristine and prednisone (CHOP) or a regimen comparable with CHOP that includes anthracyclines. Over the last few decades, an association between NHLs and autoimmune diseases has been gradually established (3). Certain studies have demonstrated that autoimmune diseases are associated with a higher risk of developing NHLs, while other findings show that the correlation between autoimmunity and lymphoproliferative tumors is bi-directional. It is hypothesized that the impaired regulation of auto-reactive or transformed lymphocytes (or a combination of the two), via programmed cell death, leads to the development of these two chronic conditions, which highlights the significance of the immune system regulation via apoptosis (3).

Pemphigus is a life-threatening autoimmune blistering disease that is classified into four major types; pemphigus vulgaris, pemphigus foliaceus, paraneoplastic pemphigus and immunoglobulin (Ig) A pemphigus (4). IgA pemphigus is characterized by tissue-bound and circulating IgA antibodies, which target cell surface components in the epidermis. This disease is histopathologically characterized by extensive epidermal infiltration by neutrophils and slight epidermal acantholysis. IgA pemphigus was proposed as a disease entity relatively recently and, to the best of our knowledge, only 60 cases have been reported to date. The male-to-female ratio of IgA pemphigus is 1:1.33 and the average age of the affected individuals is 53 years. IgA pemphigus is considered to be less life-threatening than other types of pemphigus; thus far, there have been no reports of IgA pemphigus patients who have succumbed as a direct result of the disease (5). The clinical manifestations of IgA pemphigus are flaccid blisters or pustules on normal or erythematous skin. The majority of pustules fuse and form a large ring with scales and scabs in the center, and blisters in the margin. Nikolsky’s sign is usually negative, however, specific cases that exhibited positive results have been reported. Additionally, mucous membranes are not usually affected in IgA pemphigus.

IgA pemphigus is further subdivided into the subcorneal pustular dermatosis (SPD)-type, whose target antigen is desmocollin 1, and intraepidermal neutrophilic IgA dermatosis (IEN)-type, whose target antigen is unknown although is potentially a non-desmosomal cell surface protein (9–11). The pustules localize in the subcorneal region in SPD-type IgA pemphigus, whereas they are present in the entire or mid-epidermis in IEN-type IgA pemphigus. Diagnostic direct immunofluorescence reveals IgA deposition in the epithelial prickle cell membrane and IgG or complement 3 (C3) may be deposited, however, to a lesser extent than IgA. Indirect immunofluorescence typically shows circulating IgA antibodies (7–9). Patient provided written informed consent.

## Case report

**Figure 1:**
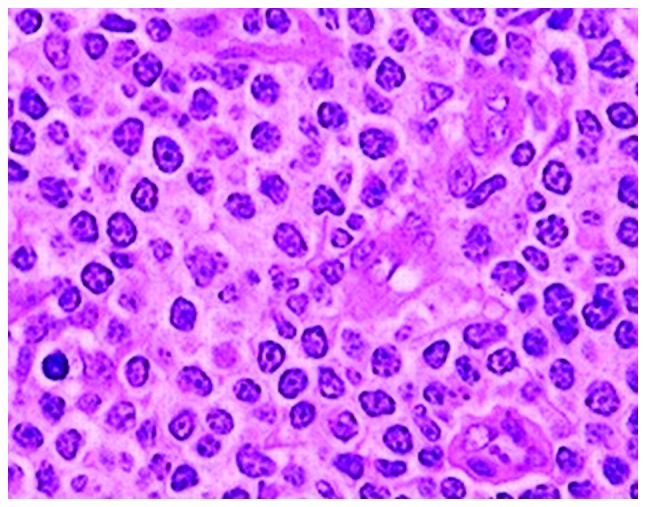
Histopathology of the lymph node in the inguinal fold with varied lymphocyte infiltration and visible nucleoli (hematoxylin-eosin stain; magnification, ×200).

**Figure 2:**
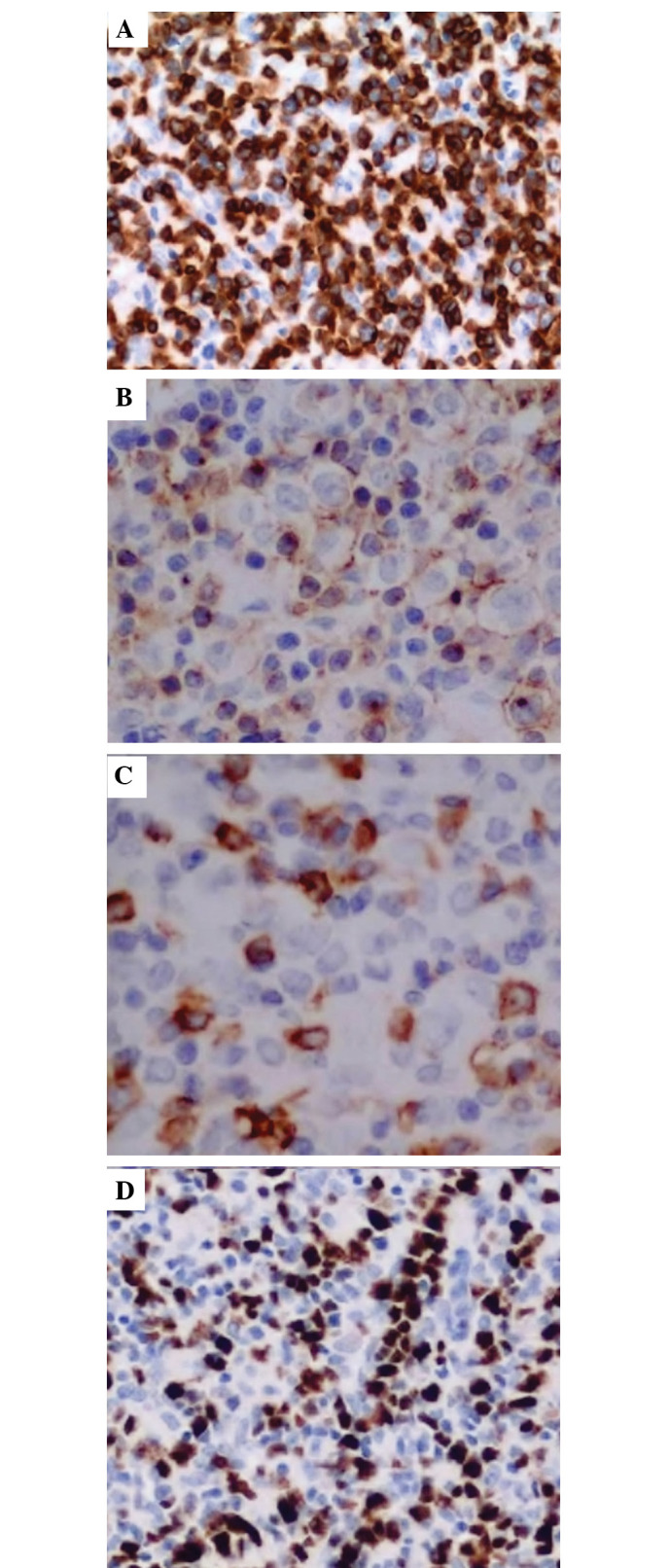
Immunohistochemical analysis of the lymph node in the inguinal fold: (A) Cluster of differentiation (CD)3-positive; (B) CD2-positive; (C) CD5-positive; and (D) Ki-67-positive (hematoxylin-eosin stain; magnification, ×200).

A 43-year-old male was admitted to the Union Hospital (Wuhan, China) in March 2012 with a two-month history of fatigue and a poor appetite. The patient also reported the presence of a palpable mass under the jaw bone one month prior to admission and did not report any symptoms of fever, night sweats or weight loss. Physical examination identified multiple swollen lymph nodes in the bilateral cervix, the supraclavicular-axillary region and the inguinal fold. The largest node measured 4.5×3 cm^2^ and was located in the right inguinal fold. Pathology of the right inguinal lymph node revealed PTCL. Immunohistochemical staining was positive for cluster of differentiation (CD)2, CD3, CD5, CD7, and CD47, and negative for CD20. Ki-67 was ~40% positive (Figs. 1 and 2A–D). The computed tomography scan performed with a contrast agent displayed a clean mediastinum, abdomen and pelvis, all without swollen lymph nodes. The bone marrow exam was normal. The patient was diagnosed with PTCL and stage IIIA disease, and received four cycles of chemotherapeutic CHOP between March 25 and May 30, 2012; the patient response was partial remission (PR). In June 2012, the B symptoms (lymphoma specific symptoms: Fever, night sweats and weight loss) reported by the patient were fever and night sweats. The lymph node in the inguinal fold had increased in size, with a tumor evaluation indicating progressive disease according to the Response Evaluation Criteria in Solid Tumors (RECIST 1.1) (12). The patient received two cycles of gemcitabine, cisplatin and dexamethasone between June 27 and July 22, 2012. The response was PR according to RECIST 1.1 (12), and the patient decided to terminate treatment. In September, re-examination showed enlarged lymph nodes in the retroperitoneal region, however, the other regions were normal. The patient received a 36-Gy total dose of radiation to the retroperitoneal region, and the response was an unconfirmed complete remission. The patient was prescribed oral thalidomide (125 mg daily) as maintenance therapy for one month.

**Figure 3:**
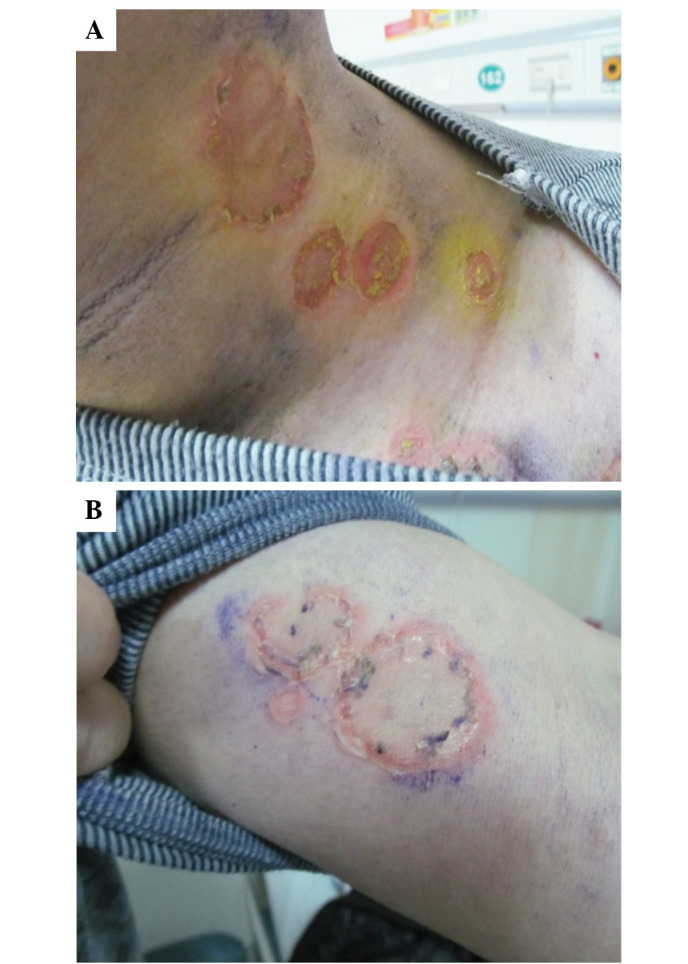
Erosion patches on the (A) left neck and (B) left arm.

**Figure 4:**
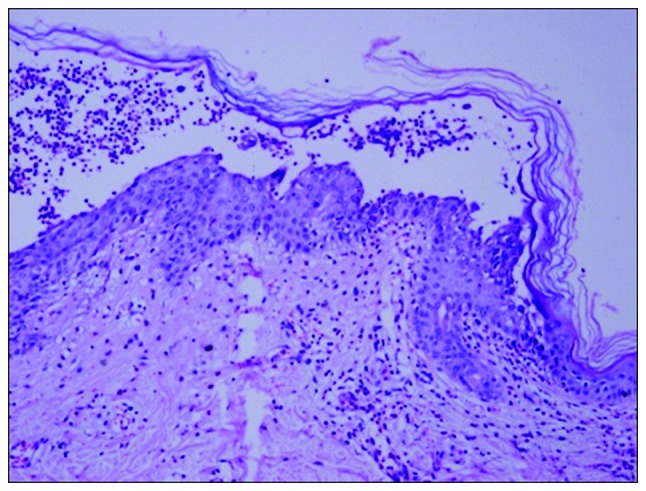
Histopathology of the skin lesion. Subcorneal pustular, marginal epidermal acantholysis and extensive neutrophilic infiltration in the epidermis (hematoxylin-eosin stain; magnification, ×100).

**Figure 5:**
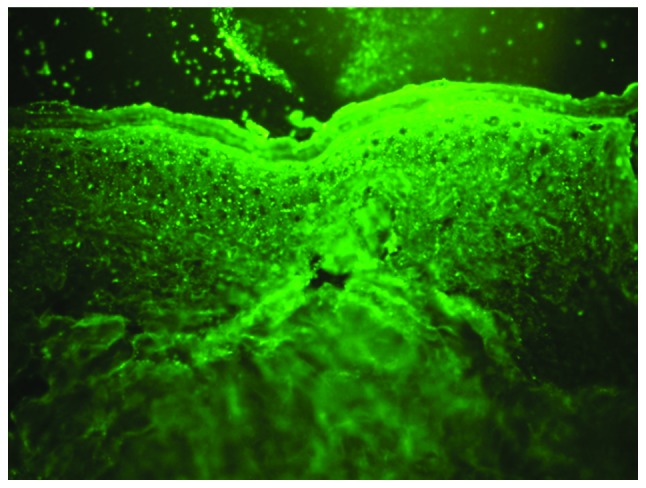
Direct immunofluorescence of the biopsy showing immunoglobulin A deposits (immunofluorescence stain; magnification ×100).

**Figure 6:**
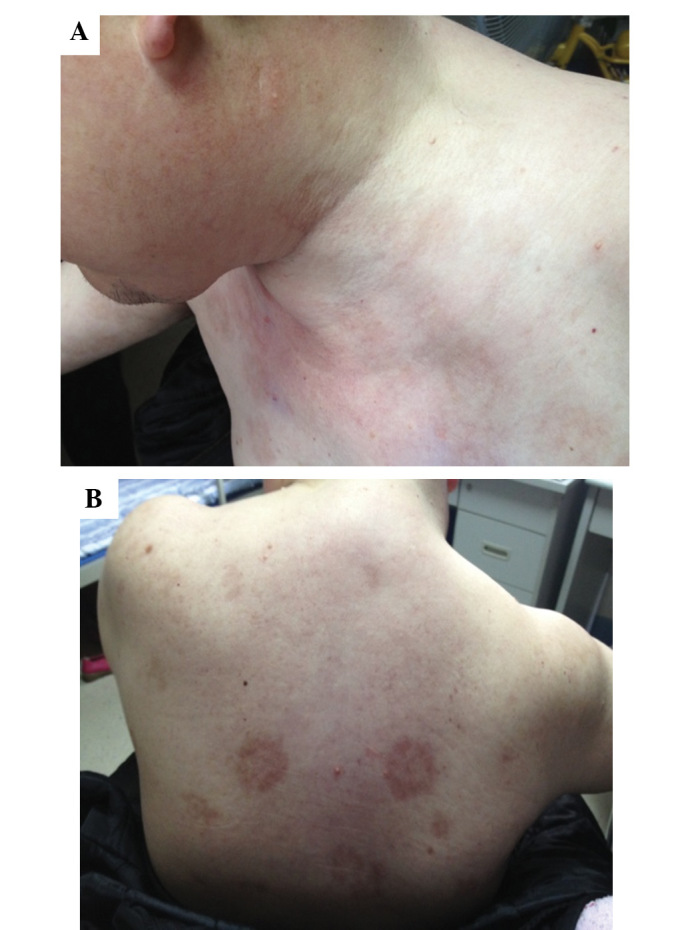
Pustular lesions disappeared subsequent to therapy, leaving scars on the (A) neck and (B) back.

Subsequent to receiving thalidomide for ~20 days, a number of blisters were found on the trunk and limbs of the patient. The patient was referred to the dermatology department where fingernail- to nut-sized eroded patches were observed on the scalp, the four limbs, the lower abdomen and the inguinal fold. The blisters did not exhibit obvious seepage. Nikolsky’s sign was positive (Fig. 3A and B) and the oral cavity mucous membranes were spared. Histopathology of the skin lesion from the abdomen showed subcorneal pustular, slight epidermal acantholysis and extensive neutrophilic infiltration in the epidermis. Lymphocyte, histiocyte and eosinophil infiltration were observed in the superficial layer of the dermis (Fig. 4). Direct immunofluorescence of the biopsy was positive for IgA and negative for IgM, IgG, C3, C4 and C1q (Fig. 5). The clinical manifestations, histopathology and direct immunofluorescence met the diagnostic criteria of IgA pemphigus. Therefore, the patient was administered with an 80-mg methylprednisolone intravenous drip for two weeks and the blisters faded (Fig. 6). The treatment was altered to prednisone for maintenance therapy with a starting dose of 80 mg and was decreased by 10 mg every two weeks. The patient is currently taking 40 mg prednisone and the lymphoma is stable. At present, there is no evidence of recurrence of the lymphoma or pemphigus in this patient.

## Discussion

IgA pemphigus is a rare disease characterized by a vesiculopustular eruption established by epidermal intercellular IgA deposition. Various cases in the literature were associated with other diseases, including IgA paraproteinemia, IgG cryoglobulinemia, lymphoma, myeloma, rheumatoid arthritis, ulcerative colitis, Crohn’s disease, lung cancer and hyperthyroidism (13,14).

The chronic inflammation characteristic of IgA pemphigus may be due to cancer progression or drug-associated immunosuppression. Pemphigus skin diseases most often complicate blood-lymphatic system disorders. For example, there is an association between paraneoplastic pemphigus and NHL. One case of chronic lymphocytic leukemia complicated by IgA pemphigus and paraneoplastic pemphigus has been reported (15). In addition, Asahina *et al* (16) reported a case of diffuse large B cell lymphoma that was complicated by IgA pemphigus without rash recurrence for one year after treatment.

The male patient with PTCL in the present study met the diagnostic criteria of IgA pemphigus as a result of clinical manifestations, histopathology and immunofluorescence assays. Histopathology showed blistered skin, subcorneal pustules and acantholysis, and direct immunofluorescence revealed IgA deposition. Subsequent to intravenous glucocorticoid therapy and prolonged maintenance treatment with prednisone, the blisters disappeared without recurrence.

Pemphigus in the present patient appeared during thalidomide maintenance therapy of the lymphoma. Thalidomide is widely administered for the treatment of blood-lymphatic system cancers due to its anti-angiogenesis effect and its dual immunomodulatory function as an immunosuppressant or immunostimulant (17). A study by Herth *et al* (18) previously concluded that thalidomide maintenance therapy drives the maturation of T cells toward a memory phenotype, however, compromises antigen-specific immunity. Additionally, the decreased frequency and function of CD8^+^ T cells has been previously described for T-cell lymphoma (19). It was hypothesized that the cause of IgA pemphigus in the present patient may have been a combination of the immunological imbalance, which was caused by the blood-lymphatic system cancer, and thalidomide-induced immunomodulatory effects. However, further evidence is required to support this assumption.

There are no recognized guidelines for IgA pemphigus management, however, the mainstay treatment of IgA pemphigus is oral and topical corticosteroids. This treatment reduces inflammation by reversing the increased permeability in capillaries and terminating neutrophil activity (20). Dapsone and isotretinoin are also effective treatments for IgA pemphigus (21,22). Adalimumab and mycophenolate mofetil, which are effective in treating classic pemphigus, have also been reported as proficient treatments (23).

The present study is the first case of PTCL complicated by IgA pemphigus in a Chinese patient. IgA-associated autoimmune diseases, including IgA pemphigus, are clinically rare. When a suspected case of IgA pemphigus is under investigation, an immediate biopsy is recommended to acquire an early diagnosis. The present case report of a patient with PTCL complicated with IgA pemphigus facilitated the unique observation of the combination of a malignant lymphoma with a particularly rare autoimmune disease. Furthermore, the present case raises questions about the putative effects of immunomodulatory maintenance therapy for individuals that are autoimmune. In conclusion, the present study demonstrates that investigation of the association between these two diseases is necessary in future studies, as this association may determine the phenomenon of neoplasia-induced autoimmunity.

## References

[1] Vose J, Armitage J, Weisenburger D, International T-Cell Lymphoma Project (2008). International peripheral T-cell and natural killer/T-cell lymphoma study: pathology findings and clinical outcomes. J Clin Oncol.

[2] Escalón MP, Liu NS, Yang Y (2005). Prognostic factors and treatment of patients with T-cell non-Hodgkin lymphoma: the M.D. Anderson Cancer Center experience. Cancer.

[3] Martin DN, Mikhail IS, Landgren O (2009). Autoimmunity and hematologic malignancies: associations and mechanisms. Leuk Lymphoma.

[4] Hashimoto T (2003). Recent advances in the study of the pathophysiology of pemphigus. Arch Dermatol Res.

[5] Yeh SW, Ahmed B, Sami N, Razzaque Ahmed A (2003). Blistering disorders: diagnosis and treatment. Dermatol Ther.

[6] Hashimoto T (2001). Immunopathology of IgA pemphigus. Clin Dermatol.

[7] Hashimoto T, Kiyokawa C, Mori O (1997). Human desmocollin 1 (Dsc1) is an autoantigen for the subcorneal pustular dermatosis type of IgA pemphigus. J Invest Dermatol.

[8] Hashimoto T, Komai A, Futei Y, Nishikawa T, Amagai M (2001). Detection of IgA autoantibodies to desmogleins by an enzyme-linked immunosorbent assay: the presence of new minor subtypes of IgA pemphigus. Arch Dermatol.

[9] Amagai M, Komai A, Hashimoto T (1999). Usefulness of enzyme-linked immunosorbent assay using recombinant desmogleins 1 and 3 for serodiagnosis of pemphigus. Br J Dermatol.

[10] Ebihara T, Hashimoto T, Iwatsuki K (1991). Autoantigens for IgA anti-intercellular antibodies of intercellular IgA vesiculopustular dermatosis. J Invest Dermatol.

[11] Beutner EH, Chorzelski TP, Wilson RM (1989). IgA pemphigus foliaceus. Report of two cases and a review of the literature. J Am Acad Dermatol.

[12] Watanabe H, Okada M, Kaji Y (2009). New response evaluation criteria in solid tumours-revised RECIST guideline (version 1.1). Gan To Kagaku Ryoho.

[13] Petropoulou H, Politis G, Panagakis P, Hatziolou E, Aroni K, Kontochristopoulos G (2008). Immunoglobulin A pemphigus associated with immunoglobulin A gammopathy and lung cancer. J Dermatol.

[14] Szturz P, Adam Z, Klincová M (2011). Multiple myeloma associated IgA pemphigus: treatment with bortezomib- and lenalidomide-based regimen. Clin Lymphoma Myeloma Leuk.

[15] Taintor AR, Leiferman KM, Hashimoto T, Ishii N, Zone JJ, Hull CM (2007). A novel case of IgA paraneoplastic pemphigus associated with chronic lymphocytic leukemia. J Am Acad Dermatol.

[16] Asahina A, Koga H, Suzuki Y, Hashimoto T (2013). IgA pemphigus associated with diffuse large B-cell lymphoma showing unique reactivity with desmocollins: unusual clinical and histopathological features. Br J Dermatol.

[17] Oxberry SG, Johnson MJ (2006). Response to thalidomide in chemotherapy-resistant cutaneous T-cell lymphoma. Clin Oncol (R Coll Radiol).

[18] Herth I, Witzens-Harig M, Beckhove P (2013). Thalidomide maintenance therapy maturates the T cell compartment and compromises antigen-specific antitumor immunity in patients with multiple myeloma. Exp Hematol.

[19] Kozako T (2011). New treatment strategy for adult T-cell leukemia targeting for anti-tumor immunity and a longevity gene-encoded protein. Yakugaku Zasshi.

[20] Camisa C, Warner M (1998). Treatment of pemphigus. Dermatol Nurs.

[21] Gruss C, Zillikens D, Hashimoto T (2000). Rapid response of IgA pemphigus of subcorneal pustular dermatosis type to treatment with isotretinoin. J Am Acad Dermatol.

[22] Ruiz-Genao DP, Hernández-Núñez A, Hashimoto T, Amagai M, Fernández-Herrera J, García-Díez A (2002). A case of IgA pemphigus successfully treated with acitretin. Br J Dermatol.

[23] Howell SM, Bessinger GT, Altman CE, Belnap CM (2005). Rapid response of IgA pemphigus of the subcorneal pustular dermatosis subtype to treatment with adalimumab and mycophenolate mofetil. J Am Acad Dermatol.

